# A Methylome‐Wide Association Study of Trajectories of Oppositional Defiant Behaviors and Biological Overlap With Attention Deficit Hyperactivity Disorder

**DOI:** 10.1111/cdev.12957

**Published:** 2017-09-20

**Authors:** Edward D. Barker, Esther Walton, Charlotte A.M. Cecil, Richard Rowe, Sara R. Jaffee, Barbara Maughan, Thomas G. O'Connor, Argyris Stringaris, Alan J. Meehan, Wendy McArdle, Caroline L. Relton, Tom R. Gaunt

**Affiliations:** ^1^ King's College London; ^2^ University of Bristol; ^3^ University of Sheffield; ^4^ University of Pennsylvania; ^5^ University of Rochester Medical Center; ^6^ National Institutes of Health

## Abstract

In 671 mother–child (49% male) pairs from an epidemiological birth cohort, we investigated (a) prospective associations between DNA methylation (at birth) and trajectories (ages 7–13) of oppositional defiant disorder (ODD), and the ODD subdimensions of irritable and headstrong; (b) common biological pathways, indexed by DNA methylation, between ODD trajectories and attention deficit hyperactivity disorder (ADHD); (c) genetic influence on DNA methylation; and (d) prenatal risk exposure associations. Methylome‐wide significant associations were identified for the ODD and headstrong, but not for irritable. Overlap analysis indicated biological correlates between ODD, headstrong, and ADHD. DNA methylation in ODD and headstrong was (to a degree) genetically influenced. DNA methylation associated with prenatal risk exposures of maternal anxiety (headstrong) and cigarette smoking (ODD and headstrong).

Oppositional defiant disorder (ODD) is defined by the ICD (WHO, 1992) and *Diagnostic and Statistical Manual of Mental Disorders* (*DSM*; American Psychiatric Association, 2013) as a recurrent pattern of defiant, disobedient, and hostile behavior beginning in childhood or adolescence. Together with attention deficit hyperactivity disorder (ADHD) and conduct disorder, ODD is one of the leading reasons for referral and continued involvement in youth services (Burke, Mulvey, & Schubert, 2015). The worldwide lifetime prevalence rate of ODD is 10% and among those with ODD, the majority meet criteria for at least one other concurrent psychiatric diagnosis, including both internalizing (depression, anxiety) and externalizing (ADHD, conduct disorder, substance use) problems (Nock, Kazdin, Hiripi, & Kessler, 2007). Beyond concurrent comorbidity, ODD in childhood is also highly predictive of a range of adult difficulties (Burke, Rowe, & Boylan, 2014).

Given its high prevalence rate and the association with a wide range of adjustment difficulties, it has been proposed that ODD may represent a complex and multidimensional psychiatric category (Stringaris & Goodman, 2009). Evidence indeed suggests that ODD can be seen as including both an *irritable* (i.e., temper outbursts, easily annoyed, angry/resentful) and a *headstrong* (i.e., argued with grown‐ups, rule violations, purposefully annoy others, blame others) subdimension (Stringaris & Goodman, 2009), which show high discriminant validity. Although these two subdimensions are correlated, numerous studies have now shown that the irritable subdimension is prospectively associated with internalizing difficulties (i.e., depression), whereas the headstrong subdimension is prospectively associated with externalizing difficulties (Whelan, Stringaris, Maughan, & Barker, 2013). As a result, the *DSM–V* (American Psychiatric Association, 2013) now recognizes the irritable and headstrong subdimensions, and a recommendation has been made for irritable as a specifier to ODD in the ICD–11 (Lochman et al., 2015).

Despite the clinical relevance of ODD and its subdimensions, however, surprisingly little is known about (a) respective biological influences and (b) the extent to which these influences are shared or distinct—both within and between disorders. For example, a number of twin studies have not only shown that ODD symptoms in general have a strong heritable basis (Barker, Cecil, Walton, & Meehan, 2017), but also that ODD shares substantial common genetic variance (Tuvblad, Zheng, Raine, & Baker, 2009) and environmental influence (Burt, Krueger, McGue, & Iacono, 2001) with ADHD and other behavior problems. Hence, genetic as well as familial and contextual influences can contribute to the comorbidity between ODD and ADHD. With regard to the ODD subdimensions, only one twin study (Stringaris, Zavos, Leibenluft, Maughan, & Eley, 2012) has examined the contribution of genetic and environmental influences and reported that irritable and headstrong subdimensions share substantial common genetic influence (*r*_g_ = .66), but little common environmental influence. Moreover, irritable shared common influence with depression (*r*_g_ = .70), whereas headstrong shared common influence with delinquency (*r*_g_ = .80). To our knowledge, no published genome‐wide association study has focused on ODD; however, a recent study attempted to contrast irritable and headstrong, but did not identify genome‐wide significant loci (Aebi et al., 2015). Candidate gene studies that have examined ODD have often targeted genetic variability in the serotonergic and dopaminergic system, which are also implicated in ADHD and conduct problems (Malmberg, Wargelius, Lichtenstein, Oreland, & Larsson, 2008). No published candidate gene studies have compared the irritable and headstrong subdimensions. Hence, although twin studies show heritable biological influence, the extent to which (a) ODD and the subdimensions share specific influences and (b) how these specific influences may associate with comorbid externalizing problems (ADHD, conduct problems) has largely not been elucidated (e.g., Harvey, Breaux, & Lugo‐Candelas, 2016).

Research has begun to demonstrate the potential of epigenetic processes for understanding biological processes that associate with child and adolescent psychiatric disorders (Mill & Heijmans, 2013). Animal models and human studies indicate that genetic and environmental effects known to coact on early psychiatric problems are likely to intersect via epigenetic modifications. Epigenetic processes are essential for normal cellular development and differentiation, and allow the long‐term regulation of gene function through nonmutagenic mechanisms (Henikoff & Matzke, 1997). Data show that epigenetic processes are responsive to both genetic and environmental influences. With regard to genetic influences, twin research has shown that DNA methylation, the most researched type of epigenetic process in humans, is highly heritable in the promoter regions of genes (Kaminsky et al., 2009), and that variability in DNA methylation over time can be partially attributable to heritable factors (e.g., between 20% and 97% across different genes; Heijmans, Kremer, Tobi, Boomsma, & Slagboom, 2007). Molecular studies also report that genetic influence on DNA methylation can be be present at birth and be somewhat stable across the life course (Gaunt et al., 2016). These methylation quantitative trait loci (mQTL) have been found to associate with gene expression and may serve as markers for genetic influence on gene regulation.

With regard to environmental effects, DNA methylation has been shown to vary as a function of numerous nutritional, chemical, physical, and psychosocial exposures (Szyf & Bick, 2013). The methylation of CpG sites, overrepresented in CpG islands and often located in the promoter regulatory regions of many genes, disrupts the binding of transcription factors and can have important effects on normal gene function, hence providing a potential mechanism for long‐term alterations (and maintenance) in phenotype (Meaney, 2010). DNA methylation is proposed as a mechanism by which exposure to early adverse conditions during critical periods of development can result in long‐term vulnerability for disease (Gluckman, Hanson, Spencer, & Bateson, 2005). To date, much of what is known about DNA methylation is based on animal research where variability in DNA methylation in response to prenatal risk exposures and teratogens has been examined in highly controlled experimental research (Roth, 2013). Animal findings are beginning to be validated in observational studies in humans using peripheral samples (i.e., blood, buccal cells). For example, prenatal maternal depression, anxiety, nutrition, and toxin exposure (e.g., cigarettes smoking) associates with changes in DNA methylation in the cord blood of newborns (e.g., Binder & Michels, 2013; Richmond et al., 2015).

It is important to note that animal studies offer the ability to examine tissue‐specific DNA methylation with central nervous system (CNS) function and to experimentally validate a casual mechanistic role in disease etiology. In living humans, the study of DNA methylation is often limited to peripheral samples, which may not necessarily be a surrogate of CNS activity or be mechanistically involved in a disease. For example, although certain studies with living children/adolescents have attempted to biologically characterize the identified DNA methylation associations by testing whether effects replicate across multiple tissues (Dadds, Schollar‐Root, Lenroot, Moul, & Hawes, 2016), associate with gene expression (Ruggeri et al., 2015), or even the structure or function of the brain (Walton et al., 2017), the mechanistic role of peripheral DNA methylation in the etiology of psychopathology is largely unknown. In fact, it is equally possible that DNA methylation can function as a noncausal biomarker of environmental risk exposure and/or stress‐related disorders. Here, differences in DNA methylation may be a consequence of disease etiology (e.g., risk exposure, genetic vulnerability, and/or psychopathology) rather than a causal mechanism within the disease process. In etiologic epidemiology, this is termed “reverse causality” (Ladd‐Acosta & Fallin, 2015). Yet even in this situation DNAm can still serve as an important biomarker of disease and have clinical utility. For example, epigenetic patterns have already been shown to be useful in cancer detection, prognosis, and even predicting response to treatment (Ladd‐Acosta & Fallin, 2015).

In humans, DNA methylation has indeed been discussed as a potential biomarker (e.g., Barker, Walton, & Cecil, 2017; Szyf, 2015) indexing not only early risk exposure(s), but also vulnerability for behavioral and/or emotional problems, both in prospective birth cohorts—starting as early as birth (Rijlaarsdam et al., 2017)—and cross‐sectional clinical samples in childhood (Dadds, Moul, Hawes, Mendoza Diaz, & Brennan, 2015), adolescence, and adulthood (Frodl et al., 2015). Some of these DNA methylation studies have targeted prespecified candidate genes, selected on the basis of known biological and functional relevance to the risk exposure and the psychiatric disorder in question. Indeed, research is beginning to examine both prenatal risk, DNA methylation at birth, and subsequent vulnerability for psychiatric disorder (Barker, Walton, et al., 2017). For example, Rijlaarsdam et al. (2017), interested in the comorbidity between conduct problems and ADHD, reported that prenatal unhealthy diet (fast food, sweets) associated with higher insulin‐like growth factor 2 gene (*IGF2*) methylation at birth (i.e., cord blood), which, in turn, associated with higher ADHD symptoms for children with early onset conduct disorder. The study focused on *IGF2* due to its role in metabolic function, placental and fetal growth, and the development of brain regions that associate with ADHD (Heijmans et al., 2008).

When the pathophysiology of a disease is known, it can be straightforward to define candidate genes. However, for complex and multiply determined disorders, such as ODD (and its subdimensions), the exact pathophysiology is not yet known, therefore candidate genes—such as *IGF2*—are not likely to explain the majority of variance of a disorder (Salvatore & Dick, 2018). Hypothesis‐free scans of DNA methylation across the genome (i.e., the methylome) allow for discovery of novel biological correlates, which can aid in the development of more accurate and holistic etiologic knowledge. A recent methylome‐wide analysis study by Walton et al. (2016), for example, reported a developmental trajectory of high ADHD symptoms (ages 7–15) associated with DNA methylation (at birth) in 13 genetic loci. Of interest, one of the methylome‐wide significant loci was linked to *ZNF544*, previously shown to associate with ADHD (Lasky‐Su et al., 2008). Walton et al. (2016) did not assess the extent to which DNA methylation loci were influenced by genetic influence and/or prenatal environmental stress exposures or if the ADHD loci associated with ODD—despite research previously described showing genetic and environmental influences on both DNA methylation (Gaunt et al., 2016; Rijlaarsdam et al., 2017) and the comorbidity ODD and ADHD (Barker, Cecil, et al., 2017).

Using prospective data drawn from a large population‐based sample, featuring DNA methylation at birth and ODD trajectories spanning childhood to adolescence, the current study had four overall research aims. First, we conducted hypothesis‐free, methylome‐wide analyses of trajectories of ODD and the subdimensions of irritability and headstrong, respectively (while controlling for each other). The second overall aim was to examine genetic overlap between DNA methylation of the ODD and the subdimensions with a recent methylome‐wide association study of trajectories of ADHD (Walton et al., 2016). In reminder, twin studies suggest that the phenotypic correlation between ODD and ADHD is substantially explained through shared genetic influence (Tuvblad et al., 2009) and phenotypic studies show that both ODD and headstrong associate with ADHD (Stringaris & Goodman, 2009). However, existing research has yet to identify specific biological pathways that might underlie the shared genetics of ODD and ADHD (Harvey et al., 2016). Furthermore, recent research suggests that epigenetic effects on the development of psychiatric disorder can be time specific, with DNA methylation at birth indexing early biological vulnerability (Cecil, Walton, et al., 2016). Walton et al. (2016) utilized the same youth as the present study, and all methylome‐wide significant associations were identified at birth, which makes for an optimal framework to compare early biological pathways shared between ODD, the ODD subdimensions, and ADHD. The third aim was to assess genetic influence (i.e., mQTL) on the methylome‐wide significant ODD and subdimension DNA methylation loci. The fourth aim was to examine associations between methylome‐wide significant ODD and subdimension loci and prenatal risk exposures. Here, we examined maternal anxiety, depression, cigarette smoking, and socioeconomic risks.

## Method

### Participants

Participants were drawn from the accessible resource for integrated epigenomics studies (ARIES, http://www.ariesepigenomics.org.uk; Relton et al., 2015), containing DNA methylation data for a subset of 1,018 mother–offspring pairs and nested within the Avon Longitudinal Study of Parents and Children (ALSPAC). ALSPAC is an ongoing epidemiological study of children born from 14,541 pregnant women residing in Avon, United Kingdom, with an expected delivery date between April 1991 and December 1992 (85% of eligible population; Fraser et al., 2013). Informed consent was obtained from all ALSPAC participants and ethical approval was obtained from the ALSPAC Law and Ethics Committee as well as Local Research Committees. The original ALSPAC sample is representative of the general population (Boyd et al., 2012). Note that the study website contains details of all the data that are available through a fully searchable data dictionary: http://www.bris.ac.uk/alspac/researchers/dataaccess/data-dictionary/. For this study, we included youth from ARIES who had available data on ODD symptomatology ratings (ages 7–13) as well as epigenetic data at birth (*n* = 671, 49% male). The cohort profile of ARIES by Relton et al. (2015) compared a selection of maternal characteristics in ARIES (*n* = 1,018) to the rest of the ALSPAC sample. ARIES versus ALSPAC mothers were more ethnically homogenous (% White: ARIES = 100% vs. ALSPAC = 97.4%), slightly older (*M*_age_: ARIES = 29.2 vs. ALSPAC = 28.2), less likely to have a manual occupation (ARIES = 14% vs. ALSPAC = 20.5%), and less likely to have smoked throughout pregnancy (ARIES = 9.7% vs. ALSPAC = 19.4%). Otherwise, the subsample was considered to be reasonably representative of the main ALSPAC population.

### Measures

ODD symptomatology was assessed via maternal ratings at ages 7, 10, and 13 years, using the well‐validated Development and Well‐Being Assessment interview (DAWBA; Goodman, Heiervang, Collishaw, & Goodman, 2011). The DAWBA was administered via computer‐based package of questionnaires, interviews, and rating techniques used to assess adolescent psychopathology based on *DSM–IV* criteria. We examined the seven symptoms of ODD that tap the irritable and headstrong subdimensions. Each question was introduced with the stem: “over the last 6 months, and as compared with other children the same age, has s/he often … .” followed by the specific clause. Response categories were 0 = no, 1 = a little more than others, 2 = a lot more than others. Following the lead of Stringaris and Goodman (2009) and as we have done in prior research (Whelan, Leibenluft, Stringaris, & Barker, 2015; Whelan et al., 2013), we defined ODD irritable by the average of the following three symptoms: (a) has temper outbursts, (b) has been touchy or easily annoyed, and (c) has been angry or resentful. ODD headstrong was defined by the average of the following four symptoms: (a) argued with grown‐ups, (b) taken no notice of rules/refused to do as she or he is told, (c) seemed to do things to annoy other people on purpose, (d) blamed others for his or her own mistakes or bad behavior.

#### Prenatal Risk Exposures

Maternal symptoms of depression and anxiety were measured by self‐reports (at 18 and 32 weeks gestation) on the Edinburgh Postnatal Depression Scale (Cox & Holden, 2003) or the Crown Crisp anxiety scale (Birtchnell, Evans, & Kennard, 1988). Cigarette smoking was assessed during pregnancy by mothers reporting on the number of cigarettes smoked per day in the first 3 months of pregnancy and the number of cigarettes smoked per day in the last 2 weeks of pregnancy. These two measures were significantly correlated (*r* = .826, *p* < .0001). Poverty was coded via the Registrar General's social class scale at 32 weeks gestation. We compared mothers in Classes IV and V (low socioeconomic status) with those in Classes I, II, and III. Age of mother (*M* = 24.34; *SD* = 4.99) was dichotomized to contrast mothers who gave birth to the study child during the teens (e.g., age 19 and younger, coded 1) with all older mothers (coded 0).

#### DNA Methylation Data

Genomic DNA (500 ng) from blood (cord at birth) was bisulfite converted using the EZ‐DNA methylation kit (Zymo Research, Orange, CA, USA). DNA methylation was quantified using the Illumina HumanMethylation450 BeadChip (HM450k; Illumina, San Diego, CA, USA) with arrays scanned using an Illumina iScan (software version 3.3.28). Samples or probes that failed quality control (> 1% probes/samples with background detection *p* ≥ .05) were excluded from further analysis. Sex checks were performed using X/Y chromosome methylation. Genotype probes on the HM450k were compared between samples from the same individual and against single nucleotide polymorphism (SNP)‐chip data to identify and remove any sample mismatches. Samples were quantile normalized using the dasen function within the wateRmelon package (version 1.4.0) in R (Vienna, Austria; http://www.R-project.org). Normalization performance was evaluated using all three testing metrics in wateRmelon (genki assessing single‐nucleotide polymorphism (SNP)‐related probes, dmrse assessing imprinted probes and seabi assessing gender differences). Methylation levels were then indexed by beta values (corresponding to the ratio of the methylated signal divided by the sum of the methylated and unmethylated signals). Probes known to be cross‐reactive or polymorphic (Chen et al., 2013; Price et al., 2013) and SNP (i.e., “*r*s”) probes were removed. We also removed participants with non‐Caucasian or missing ethnicity (based on self‐reports), leaving a total of 671 samples after quality control. Cell‐type proportions (CD8 T lymphocytes, CD4 T lymphocytes, natural killer cells, B lymphocytes, monocytes, and granulocytes) for each participant were estimated using the reference‐based approach detailed in Houseman et al. (2012).

### Analyses

#### Step 1a: ODD Trajectories

We first estimated trajectories for the irritable and headstrong subdimensions, respectively. Given that *DSM–V* includes both irritable and headstrong behaviors in the ODD symptom area (American Psychiatric Association, 2013), we created the ODD trajectory by combining the groups from the trajectories of irritable and headstrong to represent two groups of youth: those high on both subdimensions (i.e., high/high ODD group) versus those low on both subdimensions (i.e., low/low group).

Trajectories were estimated through longitudinal latent profiles using Mplus v7.11 (Muthén & Muthén, 1998–2016). This type of analysis describes classes of children who may follow different developmental patterns of ODD (e.g., high vs. low levels at differing ages). A series of models were fitted beginning with a one‐class model and moving to a five‐class model. As we estimated the different trajectory models, we assessed entropy and evaluated the overall percentage of youth estimated in the different trajectories. We took this strategy as we were ultimately interested in assessing covariate effects (i.e., methylome‐wide analyses) and therefore needed to be sure that the standard errors associated with covariate effects were precise (i.e., low in bias) and that we would have enough youth in the different trajectories to perform a methylome‐wide analysis. With regard to examining covariates in latent class models, through simulation and use of epidemiological data, Heron, Croudace, Barker, and Tilling (2015) found little detriment to assessing covariates (i.e., low bias) when using a hard classify approach with high levels of entropy (≤ .91) and high class separation (i.e., low overlap in class membership). This is important, as currently (to our knowledge) there is no software program that can both estimate mixture models and to perform bias‐adjusted methods within a methylome‐wide association analysis. Hence, as we estimated the trajectories in Mplus and then ran the methylome‐wide association analyses in *R*, we paid close attention to potential bias that could affect confidence in the results.

#### Steps 1b and 1c: Methylome‐Wide Analysis

Methylome‐wide analysis tests of the association between neonatal DNA methylation (407,462 probes) and trajectories of ODD (Step 1b) and the subdimensions (Step 1c) were performed at birth, using a general linear model. All analyses were performed in R (version 3.0.2) using the package CpGassoc (Barfield, Kilaru, Smith, & Conneely, 2012), controlling for sex, cell type, and batch effects. Methylome‐wide analyses on each subdimension were also controlled for the other subdimension. Differentially methylated probes (DMPs) were considered significant if they passed a false discovery rate (FDR) correction of *q* < 0.05. To investigate the robustness of our findings, we winsorized significant FDR‐corrected probes to reduce the influence of potential outliers (> 3 *SD*) and repeated the analysis. Winsorizing was performed using the corresponding function in the DescTools R package with default settings. Only probes that passed FDR correction and windsorizing are reported in the results.

#### Step 2: Overlap of ODD Trajectories and ADHD

We examined the overlap between loci associated with ODD (and its subdimensions) versus ADHD, based on a previous methylome‐wide analysis of ADHD in this sample (Walton et al., 2016). We first examined cross‐over in the methylome‐wide significant (i.e., FDR corrected) top hits. We then examined enriched biological pathways for genes that were associated with both phenotypes (i.e., “shared” pathways of ODD and ADHD). Using an optimized gene ontology method (see Cecil, Smith, et al., 2016) that controls for a range of potential confounds, including background probe distribution and gene size (see Table S4, for details), genes were considered “shared” if probes annotated to them were associated with both ODD and ADHD (*p* < .001 consistently across both).

#### Step 3: Genetic Influence Underlying the Top Hits

We examined the degree to which the top hits (if identified) for ODD and the irritable and headstrong subdimensions were associated with genetic variants. As our sample size was underpowered to carry out genetic analyses, we did not include in the study genetic data. Instead, we used the mQTLdb resource (http://www.mqtldb.org/) to search for known mQTLs associated with our DNAm sites of interest. The mQTLdb database contains the results of a large‐scale study based on the ARIES sample in ALSPAC (from which our sample is derived), characterizing genome‐wide significant *cis* effects (i.e., SNP within 1 million base pairs of the DNAm site) and *trans* effects (i.e., beyond ±1 million base pairs) on DNAm levels across Illumina 450k probes at five different life stages, including cord blood DNAm at birth (Gaunt et al., 2016). Here, we searched for mQTLs based on results from a genome‐wide complex trait conditional analysis, which was used to identify mQTLs with the most representative, independent effect on each DNAm site in order to account for linkage disequilibrium (Gaunt et al., 2016).

#### Step 4: Prenatal Risk Associations With Top Hits

Finally, we examined associations between ODD (and subdimension) top hits and prenatal exposures. Because of the large number of significant probes, for the purpose of this analysis, we grouped all top loci for ODD and headstrong into respective cumulative methylation risk scores, instead of testing each probe individually. Specifically, as we have done previously (Cecil, Walton, et al., 2016), we applied a method used for cumulative (polygenic) risk scores (Shah et al., 2015), where we multiplied the methylation loci by their respective standardized regression betas (i.e., weights), and then summed these together into a single DNA methylation risk score. This approach enabled us to reduce the volume of our methylation data, while the use of weights ensured that the DNA methylation loci maintained their relative predictive importance (i.e., as opposed to alternative approaches such as averaging DNA methylation levels across loci). Once calculated, we examined Pearson's bivariate correlations with prenatal maternal depression and anxiety symptoms, smoking, alcohol use, and demographic risks (e.g., poverty, teen mother).

##### Missing data

Of the 1,018 families within the ARIES resource, 914 had methylation data available at cord. Twenty‐five samples at birth failed quality control (> 1% probes/samples with background detection *p* ≥ .05) and were excluded from further analysis. From the resulting cord blood sample of 889, participants with non‐Caucasian or missing ethnicity were removed (*n* = 61). This resulted in a final total of 828 at birth. We included participants with complete data for ODD and DNA methylation. This resulted in a sample of 671 youth. We compared the 671 to the 828 of the study variables (i.e., prenatal risks and ODD symptoms) and found no significant differences.

## Results

### Step 1a: Trajectories of ODD and the Subdimensions Between Ages 7 and 13

**Figure 1:**
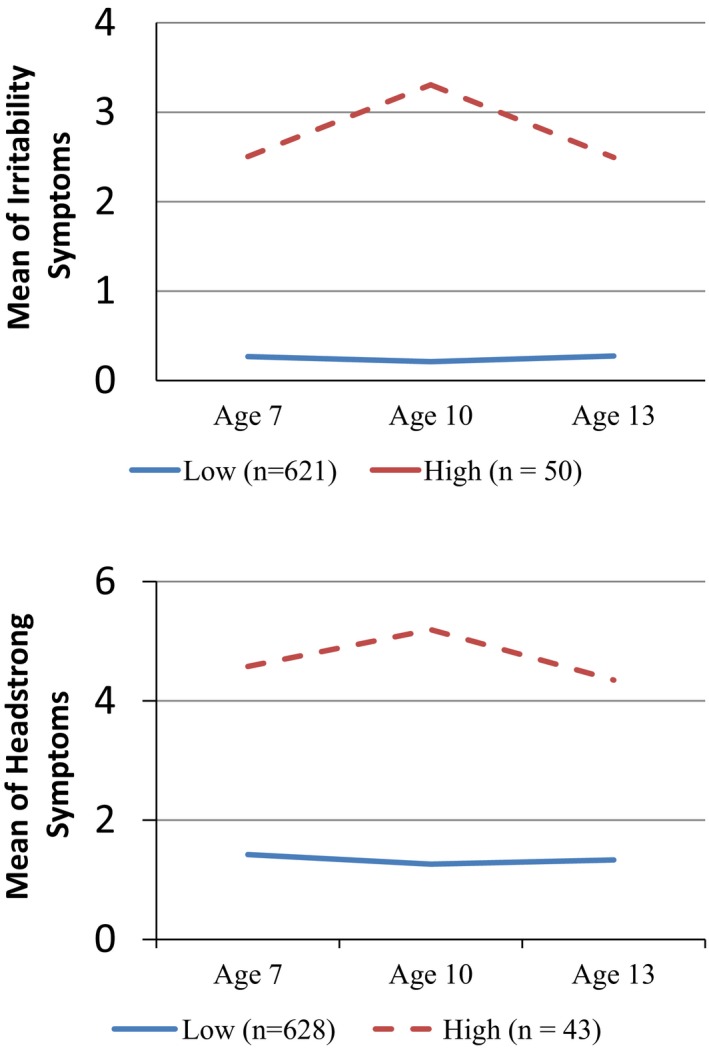
Trajectory of the subdimensions of irritable (above) and headstrong (below). [Color figure can be viewed at http://wileyonlinelibrary.com]

Trajectory analyses of the ODD subdimension symptom scores yielded a two‐trajectory solution (Figure 1) for both irritable and headstrong subdimensions (see Table S1 for fit indices). In each case, there were clearly discernible “high” and “low” groups between ages 7 and 13 years. There were *n* = 50 children in the high irritable trajectory (*n*_low‐irritable_ = 621) and *n* = 43 in high headstrong trajectory (*n*_low‐headstrong_ = 628). Approximately 50% of youth high in one subdimension were also high in the other subdimension. Hence, when we combined the youth high in both of the ODD subdimensions versus youth low in both of the subdimensions for the overall ODD trajectory, there were *n* = 23 in the high/high ODD group and *n* = 601 in the low/low ODD group.

### Step 1b: Methylome‐Wide Analysis of the Overall ODD Trajectories

**Figure 2:**
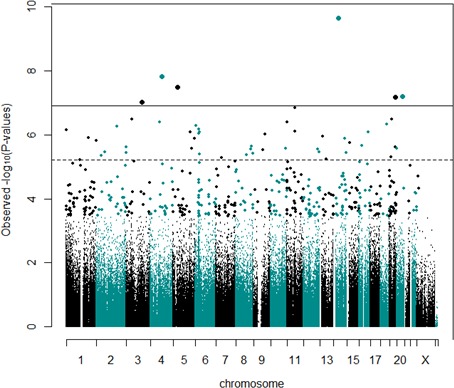
Manhattan plot of methylome‐wide results on high oppositional defiant disorder (ODD) trajectory. CpG chromosome positions are plotted against −log10 *p* values. The dotted line indicates false discovery rate (FDR)‐corrected significance threshold, the solid line indicates Bonferroni‐corrected level of significance. Results were derived using a general linear model between DNA methylation (407,462 probes at birth, cell type, and batch and sex corrected) and ODD trajectories. Only FDR‐corrected probes that also survived winsorizing are reported in the tables and followed forward. [Color figure can be viewed at http://wileyonlinelibrary.com]

We identified 30 DMPs between high versus low overall ODD trajectories after FDR correction (*q* < 0.05; Table 1 and Figure 2). There was little evidence of inflation of test statistics (λ = 1.003). Additionally, all reported probes remained significant after winsorizing to reduce the influence of outliers. Absolute mean percent methylation difference between the high and low trajectory groups for the 30 DMPs passing FDR correction was 2.5% (range = 5%–1%), which are small in effect size difference.

**Table 1:** FDR‐Corrected Probes That Associate With Oppositional Defiant Disorder Trajectory, Ranked by Birth *p* Values

High trajectory of irritability and headstrong (601 low vs. 23 high)											
Probe	Gene	Chr	Position	*F*	*p*	*q*	Mean ± *SD*		% Difference	Hedge's *g*	mQTL
							Low irritability/headstrong (*n *= 601)	High irritability/headstrong (*n* = 23)			
cg14867569	*NKX2‐1*	14	36989454	41.64	2.21E‐10	9.014E‐05	0.04 ± 0.01	0.05 ± 0.03	1	1.37	
cg08362313	*AMACR*	5	34007945	31.32	3.28E‐08	4.46E‐03	0.05 ± 0.00	0.06 ± 0.03	1	1.19	—
cg19478343	*KCNG1*	20	49620679	30.02	6.22E‐08	0.01	0.95 ± 0.02	0.92 ± 0.07	3	1.16	—
cg03626857	*ZNF227*	19	44716492	29.93	6.49E‐08	0.01	0.08 ± 0.01	0.09 ± 0.04	2	1.16	—
cg04725041	*MACROD1*	11	63906084	28.34	1.42E‐07	0.01	0.91 ± 0.03	0.88 ± 0.08	4	1.14	—
cg06088032	*ZMYND10*	3	50383227	26.71	3.19E‐07	0.01	0.08 ± 0.01	0.09 ± 0.05	2	1.09	—
cg17921484	*HAPLN4*	19	19369327	26.65	3.28E‐07	0.01	0.87 ± 0.04	0.82 ± 0.10	5	1.10	—
cg20528583	*FGF5*	4	81187610	26.28	3.95E‐07	0.01	0.05 ± 0.01	0.07 ± 0.05	1	1.09	*trans* (rs4789812; chr17)
cg04099673	*WDR7*;* TXNL1*	18	54318390	25.97	4.61E‐07	0.02	0.03 ± 0.01	0.05 ± 0.05	1	1.08	—
cg18670258	*[BTNL2]*	6	32383424	24.84	8.11E‐07	0.02	0.08 ± 0.02	0.10 ± 0.04	2	1.06	—
cg25500080	*PCDHA1*	5	140346199	24.83	8.14E‐07	0.02	0.09 ± 0.01	0.11 ± 0.04	2	1.06	—
cg07843027	*TMEM170A*	16	75498835	24.80	8.24E‐07	0.02	0.07 ± 0.01	0.09 ± 0.08	2	1.06	—
cg21107549	*ZNF311*	6	28979307	24.57	9.24E‐07	0.02	0.23 ± 0.04	0.28 ± 0.15	5	1.06	—
cg00671534	*GPR12*	13	27316496	24.18	1.12E‐06	0.02	0.86 ± 0.04	0.82 ± 0.10	5	1.04	*cis* (rs66772409)
cg14257449	*IVNS1ABP*	1	185285846	23.98	1.24E‐06	0.02	0.06 ± 0.01	0.07 ± 0.02	1	1.05	—
cg22081933	*GABRA5*	15	27128909	23.29	1.76E‐06	0.03	0.84 ± 0.04	0.80 ± 0.06	4	1.04	—
cg18672446	*USP31*	16	23160676	22.89	2.15E‐06	0.03	0.08 ± 0.02	0.11 ± 0.13	3	1.02	—
cg12570942	*DTYMK*	2	242626270	22.66	2.41E‐06	0.03	0.08 ± 0.01	0.09 ± 0.05	1	1.02	—
cg02444300	*PLA2G4C*	19	48613831	22.64	2.43E‐06	0.03	0.07 ± 0.01	0.08 ± 0.03	1	1.01	—
cg24923860	*TCOF1*	5	149765871	22.50	2.60E‐06	0.03	0.96 ± 0.02	0.94 ± 0.09	3	1.01	—
cg17888985	*[CTBP2]*	10	126898290	22.50	2.61E‐06	0.03	0.18 ± 0.04	0.22 ± 0.07	4	1.01	—
cg09998801	*PRKAB1*	12	120105695	22.39	2.75E‐06	0.03	0.06 ± 0.01	0.07 ± 0.02	1	1.00	—
cg09717987	*WDR92*;* PNO1*	2	68384793	21.96	3.42E‐06	0.04	0.08 ± 0.02	0.10 ± 0.05	2	1.00	—
cg14555045		12	128318117	21.94	3.46E‐06	0.04	0.89 ± 0.03	0.86 ± 0.07	3	1.00	—
cg22535628		2	242879655	21.82	3.67E‐06	0.04	0.89 ± 0.03	0.87 ± 0.06	3	0.99	—
cg04165845		14	101696245	21.80	3.71E‐06	0.04	0.88 ± 0.05	0.83 ± 0.08	5	0.99	*cis* (rs72704926)
cg20519035	*FAM83H*	8	144811238	21.75	3.80E‐06	0.04	0.83 ± 0.03	0.80 ± 0.06	3	0.99	—
cg24794107	*SLC39A7*;* RXRB*	6	33167627	21.67	3.96E‐06	0.04	0.05 ± 0.01	0.07 ± 0.04	1	0.99	—
cg20705804	*HECW1*	7	43590176	21.12	5.22E‐06	0.04	0.99 ± 0.01	0.98 ± 0.02	1	0.98	—
cg08471972	*FAM124A*	13	51844375	20.91	5.80E‐06	0.05	0.85 ± 0.03	0.82 ± 0.10	3	0.97	—

Note

Chr = chromosome; *p* = uncorrected *p* value; *q* = FDR‐corrected value; mQTL = methylation quantitative trait loci; FDR = false discovery rate.

Cg14867569, the most significant DMP (*p *=* *2.21E‐10; *q* = 9.01E‐05), was hypermethylated in the high overall trajectory, and is annotated to *NKX2‐1*, a gene involved in the regulation of thyroid‐specific genes (Iwatani, Mabe, Devriendt, Kodama, & Miike, 2000; Veneziano et al., 2014). Other DMPs of interest were located in genes such as *KCNG1* (cg19478343; *p *= 6.22E‐08; *q* = 0.01), coding for a voltage‐gated potassium channel (Gutman et al., 2005), and *GABRA5* (cg22081933; *p *=* *1.76E‐06; *q* = 0.03), a GABA A receptor (Wingrove et al., 1992). Also of interest was a probe associated with several genes in the *PCDHA* family, which are involved in forming cell–cell connections in the brain (Wu & Maniatis, 1999). For further details, see Table S8.

### Step 1c: Methylome‐Wide Analysis of Irritable and Headstrong ODD Subdimensions

To investigate potential associations specific to each ODD subdimension, we carried out a methylome‐wide analysis on each dimension, separately. While no probe associated with irritability after FDR correction (Table S2), 10 probes were prospectively associated with headstrong after correction (Table 2). Absolute mean percent methylation difference between the high and low trajectory groups for the 10 DMPs passing FDR correction was 2.1% (range = 4%–1%), which are small in effect size difference. Only one probe—cg19478343, linked to *KCNG1*—was also associated with the high ODD trajectory. Table S3 contains functional information for loci associated with the headstrong and ODD trajectories.

**Table 2:** FDR‐Corrected Probes That Associate With Oppositional Defiant Disorder Headstrong Subdimension, Ranked by Birth *p* Values

Headstrong (low = 628, high = 43)—simple Epigenome‐Wide Association controlling for irritability											
Probe	Gene	Chr	Position	*F*	*p*	*q*	Mean ± *SD*		% Diff	Hedge's *g*	mQTL
							Low headstrong (*n *= 628)	High headstrong (*n *= 43)			
cg21633052	C4orf38	4	184018637	42.54	1.37E‐10	5.56E‐05	0.09 ± 0.01	0.10 ± 0.04	2	0.93	—
cg09482780	KDM6B	17	7756609	30.06	5.95E‐08	0.01	0.95 ± 0.02	0.93 ± 0.07	2	0.79	—
cg05509777	C11orf21; TSPAN32; C11orf21	11	2322517	29.85	6.59E‐08	0.01	0.07 ± 0.02	0.09 ± 0.04	2	0.79	*cis* (rs2521269)
cg07150166	LCLAT1	2	30669952	29.82	6.69E‐08	0.01	0.07 ± 0.03	0.10 ± 0.07	3	0.80	*cis* (rs829657)
cg01681367	SPN	16	29676071	28.96	1.03E‐07	0.01	0.05 ± 0.01	0.06 ± 0.05	1	0.78	—
cg19478343	KCNG1	20	49620679	28.49	1.29E‐07	0.01	0.95 ± 0.02	0.92 ± 0.07	3	0.99	—
cg09057954		21	32935546	26.24	3.96E‐07	0.02	0.90 ± 0.02	0.88 ± 0.07	1	0.59	—
cg19414383		1	17528238	25.73	5.09E‐07	0.02	0.27 ± 0.05	0.31 ± 0.07	4	0.78	—
cg23137936		11	45724816	24.94	7.58E‐07	0.03	0.89 ± 0.02	0.87 ± 0.05	2	0.74	—
cg19542816	HOXD1	2	177053295	24.52	9.33E‐07	0.03	0.07 ± 0.01	0.08 ± 0.01	1	0.68	—

Note

Chr = chromosome; *p* = uncorrected *p* value; *q* = FDR‐corrected value; mQTL = methylation quantitative trait loci; FDR = false discovery rate.

### Step 2: Overlap Between ODD, Headstrong, and ADHD

Irritable was not examined further due to the lack of methylome‐wide significant loci. We did examine the overlap in DNA methylation of loci associated with ODD and headstrong and ADHD in two ways: (a) overlap in top FDR corrected hits between the two phenotypes, and (b) a gene ontology overlap analysis based on loci that were significant at *p *<* *.01 for each construct.

**Figure 3:**
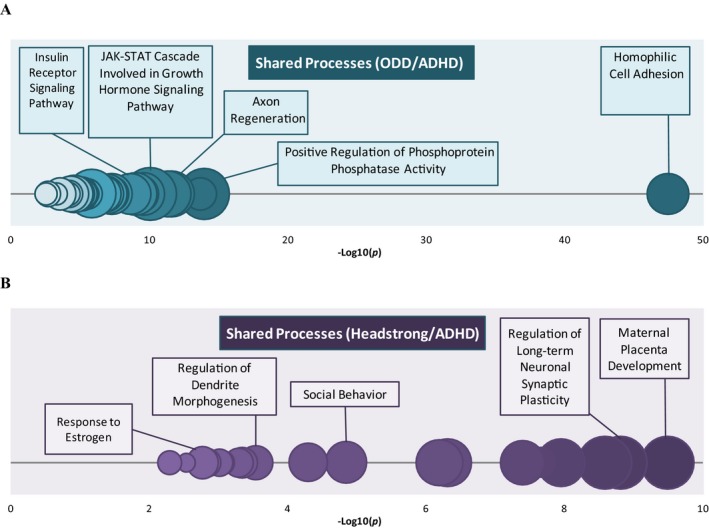
Overlap analyses for oppositional defiant disorder (ODD) and attention deficit hyperactivity disorder (ADHD; A) and headstrong and ADHD (B). Significantly enriched biological processes for genes shared between ODD, headstrong, and ADHD, based on gene ontology (GO) analysis. Circles represent GO terms that survive false discovery rate correction. The *x‐*axis represents −log(10) *p* values. The opacity of the circles indicates level of significance (darker = more significant). The size of the circles indicates the percentage of genes in our results for a given pathway compared to the total number of genes in the same pathway (i.e., larger size = larger %; range = 3.39%–20%). [Color figure can be viewed at http://wileyonlinelibrary.com]

There were no overlapping probes (or genes) between ODD, Headstrong, and ADHD above FDR correction (see Table S4). With respect to the biological pathways analysis, we found that 108 genes (see Table S5 for complete list) overlapped between ODD and ADHD (*p *<* *.001 across both phenotypes). The most enriched biological process (see Figure 3A) shared between ODD and ADHD related to processes such as cell adhesion (*p* = 3.32E‐48). Core genes in that pathway included *FAT4* and other members of the protocadherin family, important in the establishment and function of specific cell–cell connections in the brain (Wu & Maniatis, 1999). Other pathways of interest included axon regeneration (*p* = 2.51E‐10), hormone and insulin signaling pathways (*p* = 1.25E‐07), and a pathway related to insulin receptor signaling (*p* = 3.21E‐06).

With regard to headstrong, we found that 57 genes (see Table S6 for complete list) overlapped with ADHD (*p *<* *.001 across both phenotypes). The most enriched biological process (see complete list Table S6) shared between headstrong and ADHD (see Figure 3B) related to maternal placental development (*p* = 3.19E‐10), followed by regulation of long‐term neuronal synaptic plasticity (*p* = 1.54E‐09). Of note, enriched cellular components included postsynaptic density (*p* = 7.10E‐07), while the most enriched molecular function related to glutamate receptor binding (*p* = 3.83E‐05). Three genes featured most predominantly among these pathways: (a) *GRIN1*, encoding a member of the ionotropic class of glutamate receptors, implicated in learning and memory, as well as intellectual disability and schizophrenia; (b) *CAMK2B*, a gene also involved in glutamate signaling, synaptic plasticity, and dendritic remodeling; and (c) *SHANK2*, involved in the organization and structure of excitatory synapses, including glutamate receptors, which has been previously associated with autism and psychosis susceptibility (Homann et al., 2016).

We also examined the overlap of the 108 ODD–ADHD genes and 57 headstrong–ADHD genes. A total of 15 genes (see Table S7 for complete list) were common between ODD, headstrong, and ADHD. These 15 genes account 14% of the total ODD–ADHD overlap and 20% of the total headstrong–ADHD overlap.

### Step 3: Genetic Influence Underlying Top Hits for ODD and Headstrong

DNA methylation sites identified for ODD (see Table 1) and headstrong (see Table 2) were carried forward to explore associations with potential genetic influences. Based on mQTLdb search, we found that 3 of the 30 top hits for ODD and 2 of the 11 hits for headstrong were associated with mQTLs, suggesting that DNA methylation levels across these sites are likely to be influenced by known genetic polymorphisms, at birth.

### Step 4: Prenatal Risk Associations

Bivariate correlations between prenatal risk exposures and the cumulative DNA methylation scores for ODD and headstrong are shown in Table 3. As can be seen, for ODD, smoking during pregnancy positively associated with the cumulative DNA methylation score (cord blood at birth). For headstrong, maternal anxiety symptoms (18 weeks), smoking, and teen pregnancy associated positively with the cumulative DNA methylation score (cord blood at birth). Table S8 contains the correlations for prenatal exposures by individual ODD and headstrong loci.

**Table 3:** Associations Between Prenatal Exposures and Cumulative DNA Methylation at Birth

	Prenatal environment							
	Maternal depression 18 weeks	Maternal depression 32 weeks	Maternal anxiety 18 weeks	Maternal anxiety 32 weeks	Smoking (number per day, months 1 through 3)	Smoking (number per day last 2 weeks)	Poverty	Teen pregnancy
Cum DNA methylation: ODD	−0.041	−0.007	−0.002	−0.040	0.087 *p* = .025	0.09242 *p* = .017	0.315	0.027
Cum DNA methylation: headstrong	0.043	0.046	0.116 *p* = .005	0.051	0.094 *p* = .016	0.118 *p* = .002	0.001	0.100 *p* = .011

Note

Cum = cumulative; *p *= statistical probability; ODD = oppositional defiant disorder.

## Discussion

In the current study, we aimed to examine (a) methylome‐wide associations (at birth) for trajectories of ODD and the subdimensions of headstrong and irritable (ages 7–13), (b) potential biological overlaps—as indexed by DNA methylation—between these ODD trajectories and a recent methylome‐wide study of trajectories of ADHD (Walton et al., 2016), and (c) genetic and (d) prenatal influences on the DNA methylation. Prior to discussing findings relevant to these research aims, we first discuss the ODD trajectories with respect to the existing literature.

We identified two trajectories (high and low) for both irritable and headstrong subdimensions of ODD. Each high trajectory constituted 7%–8% of the sample (*n*_irritable_ = 50; *n*_headstrong_ = 43). Children classified into the high trajectory in both irritable and headstrong made up 4% of the sample and formed our ODD group (*n*_ODD_ = 23). The shapes of our trajectories and proportions of youth estimated to follow them largely fall in line with previous ODD symptom studies that have used similar analytic techniques. For example, van Lier, Van der Ende, Koot, and Verhulst (2007), using a large (*n* = 2,076) cohort from the Netherlands, identified 6% of the sample followed a similar high and chronic trajectory (4–18 years of age). Ezpeleta, Granero, de la Osa, Trepat, and Domènech (2016) followed Spanish preschoolers (*n* = 622; ages 3–5) and reported that 3.5% of the sample were in a high and chronic trajectory of ODD irritability. Of note, these trajectory studies support epidemiological results that also have identified an early onset and stable group of children showing high ODD symptomatology (Costello, Mustillo, Erkanli, Keeler, & Angold, 2003).

Our first novel finding is that we extended previous ODD trajectory studies by examining methylome‐wide significant associations. We note that our data preclude the possibility of clarifying whether these DNA methylation associations may reflect non‐CNS surrogate biomarkers versus a surrogate CNS mechanistic process. For a CNS surrogate interpretation of results, we would need access to multiple tissues, gene expression, and brain structure and/or function, as well as the application of more sophisticated causal methods. That said, in total, we identified 30 methylome‐wide significant loci for ODD, 11 for headstrong, but none for irritable. For ODD, many of the top probes related to genes such as *KCNG1*,* GABRA5*, and *WDR7*. These genes are involved in neurotransmitter and cell signaling. Also of note is a probe located in a region coding for several protocadherin genes, which are highly expressed in the brain and most likely play a critical role in the establishment and function of specific cell–cell connections in the brain (Hayashi et al., 2014). Of interest, the protocadherin superfamily of genes had previously been identified in a study that examined the impact of early‐life poverty on adult DNA methylation (Borghol et al., 2012). An unexpected result was the lack of overlap between the FDR significant loci associated with the trajectories of ODD and headstrong (in addition to the low overlap in genes shared in ODD–ADHD and headstrong–ADHD). Given that the difference between the two trajectories is that the ODD group contains the youth with both high headstrong and high irritable, headstrong in the presence of irritability appears to have different risks from headstrong in the absence of irritable. Indeed, only one probe—linked to *KCNG1*—associated with both the high ODD trajectory and the high headstrong trajectory. *KCNG1* may have a role in enhanced gene expression of voltage‐dependent ion channels during neural differentiation of stem cells in pregnancy (Park, Kang, & Hong, 2013).

The second aim of this study was to examine the potential biological overlap of ODD, the ODD subdimensions, and ADHD at birth. In reminder, the strong association between ODD and ADHD is (in part) explained through common genetic influence (Tuvblad et al., 2009). Because irritable did not have methylome‐wide significant loci, we focused on the ODD and headstrong trajectories. While we did not identify an overlap in methylome‐wide significant loci, we did identify shared biological pathways with ADHD for both ODD (108 genes) and headstrong (57 genes); however, as stated earlier, these biological pathways were relatively independent of each other as 15 genes in total overlapped between ODD, headstrong, and ADHD. For ODD and ADHD, 108 genes overlapped at *p* < .0001, and these related to neuronal cell adhesion as well as hormone and insulin signaling pathways. Importantly, a cluster was again identified involving protocadherin genes, suggesting the importance of this family to both ODD and ADHD. These genes play a pivotal development of the neural circuitry as well as in mature synaptic function (Redies, Hertel, & Hübner, 2012). Protocadherin genes associate with neuropsychiatric disorders, such as schizophrenia, autism, and bipolar disorder (Hayashi et al., 2014; Pedrosa et al., 2008). For headstrong and ADHD, 58 genes overlapped at *p* < .001, and these related to biological pathways implicated in maternal placental development, regulation of long‐term neuronal synaptic plasticity, and enriched molecular function related to glutamate receptor binding. Here, a cluster of glutamate receptor genes was identified. These genes have been implicated in intellectual disability, schizophrenia, autism, and psychosis (Berkel et al., 2010; Homann et al., 2016). Although potentially pointing toward shared early biological vulnerability for ODD, headstrong, and ADHD, the present results should be considered preliminary and are in need of replication and extension.

Our third aim was to assess genetic influence on the methylome‐wide significant loci. DNA methylation of 3 of the 30 loci for ODD and 2 of the 10 loci for headstrong were likely genetically influenced. These were mainly *cis‐*acting genetic influences that occur close to the methylation site rather than *trans*‐acting influences that occur elsewhere farther in genomic location. Although we assessed only cord blood at birth, Gaunt et al. (2016) have estimated that although levels of DNA methylation can vary across development, genetic influences are stable (average SNP heritability of DNA methylation ~0.20). Hence, an examination of genetic influences is likely important for studies that assess associations between environmental exposures and DNA methylation. As many studies (such as the present one) may not have the power to perform actual SNP interactions, the mQTLdb database (http://www.mqtldb.org/) may be of high value, as this online resource allows investigators to search the results of a large‐scale study to characterize genome‐wide significant *cis* and *trans* effects on Illumina 450k DNA methylation probes (Gaunt et al., 2016).

The fourth aim was to assess association between prenatal risk exposures and the methylome‐wide significant loci. We found that proximal exposures such as maternal anxiety, smoking, and teen pregnancy associated with the cumulative DNA methylation score for headstrong (based on 10 top hits) rather than the more distal exposure of poverty. This finding may support research that shows that DNA methylation (in cord blood) is responsive to environmental influences, with effects related to toxins in cigarettes being highly replicated across epigenetic studies (Richmond et al., 2015). Prenatal anxiety has also previously been associated with DNA methylation in cord blood, presumably through stress hormones affecting the regulation of placental barrier genes (Monk et al., 2016). Of interest, both prenatal maternal smoking and internalizing problems have been associated with child externalizing problems in genetically sensitive designs (Salatino‐Oliveira et al., 2016).

It is of interest, in comparison to headstrong, that associations between the prenatal exposures and the cumulative DNA methylation score for ODD were limited to cigarette smoking. While the mixed findings may be due to different loci, or even to combining 30 loci into a cumulative score, it may also be due to the influence of irritability in the overall ODD trajectory. In the present study, we found no methylome‐wide significant loci (at birth) for irritable. This finding may support our previous research where we did not identify a direct association between prenatal maternal stress and the irritable subdimension examined here (Whelan et al., 2015). Rather, prenatal maternal depression associated with higher symptoms of ODD irritable through increased difficult child temperament (Whelan et al., 2015).

Findings should be interpreted in light of a number of limitations. First, the current study was based on a modestly sized population‐based sample of youth. In future, it will be important to test the robustness of findings using other epidemiological cohorts. Second, findings were based on DNA methylation from peripheral samples. Therefore, research will be needed to establish the relevance of the identified markers to brain function. Future studies incorporating imaging data will be important for establishing whether these markers associate with structural or functional alterations in ODD‐relevant neural pathways (e.g., related to reward processing, impulse control). Third, despite the fact that we identified prospective associations between DNA methylation and ODD, it is not possible to establish causality, as associations could reflect the contribution of confounding genetic and environmental influences. Fourth, the study focused exclusively on DNA methylation; other epigenetic processes (e.g., histone modifications) are likely to be important influences on the development of ODD and the irritable and headstrong subdimensions. Fifth, the identification of unique versus shared biological pathways linked to ODD and ADHD were based on gene ontology analyses, which can be susceptible to bias (Timmons et al., 2015), and consequently will necessitate replication.

DNA methylation has received attention as a mechanism that can help explain vulnerability for disease (Meaney, 2010; Szyf & Bick, 2013). We focused on variation in DNA methylation in cord blood at birth, and highlighted both genetic influence and environmental associations during pregnancy. We identified prospective associations with ODD and headstrong, and also that ODD shared certain biological pathways with ADHD. Although promising, this evidence is currently preliminary and in need of replication. Consequently, findings should be interpreted with caution and considered more as well‐grounded hypotheses for further investigation. Furthermore, as we were not able to biologically characterize the DNA methylation associations (e.g., across multiple tissues, in gene expression, or in brain structure or function) or establish causality via approaches that integrate genetic proxies for methylation (e.g., epigenetic Mendelian randomization; Relton & Davey Smith, 2012), the present results are best interpreted as non‐CNS surrogate biomarker associations. Nevertheless, the present findings may be important in pinpointing specific DNA methylation markers for further investigation.

## Supporting information


**Table S1.** Oppositional Defiant Disorder (ODD) Trajectory Fit Statistics
**Table S2.** Top Differentially Methylated Sites Between Children Following a High (*n* = 50) Versus Low (*n* = 621) Trajectory of Irritability
**Table S3.** Gene Functions
**Table S4.** List of Genome‐Wide Significant (*q* < 0.05) Differentially Methylated Probes Associated With Oppositional Defiant Disorder (ODD) and Attention Deficit Hyperactivity Disorder (ADHD)
**Table S5.** Enriched Gene Ontology (GO) Pathways Shared Between Oppositional Defiant Disorder (ODD) and Attention Deficit Hyperactivity Disorder (ADHD; *n* = 103 Genes, *p* < .001 Across Both Phenotypes)
**Table S6.** Enriched Gene Ontology (GO) Pathways Shared Between Headstrong and Attention Deficit Hyperactivity Disorder (ADHD; *n* = 59 Genes, *p* < .001 Across Both Phenotypes)
**Table S7.** Genetic overlap between ADHD and and overall ODD and Headstrong
**Table S8.** Correlations between individual probes and prenatal risk exposures
